# Drugging the undruggable: Ross Cagan interviews Kevan Shokat

**DOI:** 10.1242/dmm.049468

**Published:** 2022-03-02

**Authors:** Ross Cagan, Kevan Shokat

**Figure:**
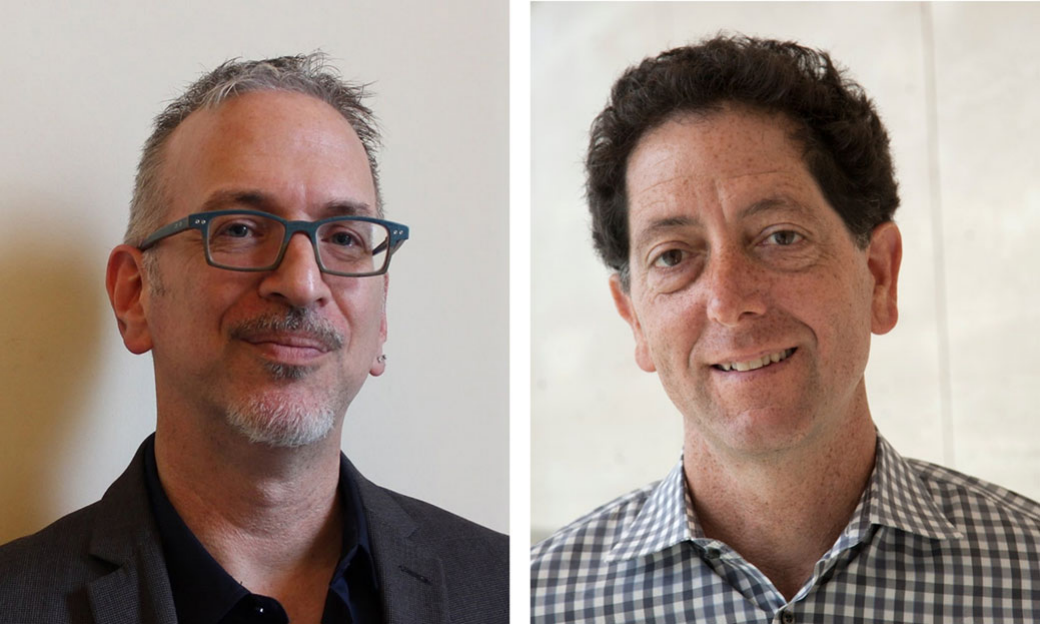
Ros Cagan (left) and Kevan Shokat (right)

Professor Kevan Shokat is a pioneer in the development of chemical tools for novel drug and drug target discovery. He has made breakthrough discoveries when targeting KRAS, PI3K and mTOR, all of which have enabled profound advances in the treatment of cancer and neurodegenerative diseases. Kevan challenged dogma when he ‘drugged the undruggable’ by successfully developing the first covalent inhibitor of mutated KRAS and, in an oncology landmark, this inhibitor has recently been approved for the treatment of non-small cell lung cancer.

Initially, Kevan pursued his passion for chemistry receiving a BA from Reed College and continuing with a PhD at UC Berkeley with Peter Schultz. He then shifted disciplines as a postdoc into the realm of immunology with Christopher Goodnow at Stanford University. Currently, he is a professor at University of California at San Francisco, where he has become an icon and fixture. He has co-founded many companies that have had remarkable success, won a plethora of awards and is fellow of several prestigious research foundations.

Professor Ross Cagan, Regius Professor of Precision Medicine at University of Glasgow, as well as an Editor for Disease Models & Mechanisms, is renowned in the fields of oncology and fly genetics. Ross investigates and therapeutically targets KRAS mutants in cancer and RASopathies. He also pioneered the ‘patient avatar’ technology in *Drosophila*, a key tool for personalized medicine. Ross and Kevan have previously collaborated to potentiate their expertise and enhance successful translation of therapies into the clinic. In this interview, Ross and Kevan discuss how they have blurred the boundaries between biology and chemistry, and examine the key to a successful collaborative project. They also delve into how young researchers can adapt to the evolving academic landscape and how all scientists can strive to effectively communicate their work to the public to better serve society.

**Ross:** We first connected through your postdoc, Arvin Dar, as my lab had stepped into Src inhibitors, and Arvin was working on this in your lab. I was then at a meeting in Stanford and I wanted somebody to work with, who could turn genetics into chemistry, and everyone suggested talking to you. When we met, I was very impressed by your understanding of fly genetics and how you could turn that knowledge into the evolution of drugs. You dived into the biology of it. **How did you become so comfortable speaking with biologists? Do you see this as a fundamental part of who you are, as a chemical geneticist? And how is that different, for example, from drug development?**

**Kevan:** I became really interested in biology, during my PhD with Pete [Schultz], because he was one of the first chemists to think very deeply about biology. Not biological problems, like biologists think of them, but biological opportunities, like a chemist would look at them. So, when I worked on catalytic antibodies, and learned about B cells, it prompted me to go to Chris [Goodnow]'s lab and work on B cell tolerance. That's where I thought about genetics, because Chris set up an unbelievable system using genetics to make a monoclonal B cell repertoire. These were elegant systems that use genetics to create this perfect microscope to look at an amazingly complex biological question, like the antibody repertoire and how this diversity is kept in check so as not to attack ‘self” – and it really took hold.

**Ross:** There's certainly a skill that you have, you make people feel comfortable to work with and, in the type of chemistry you do, it's fundamental that you're able to work well with others. I know that you've had people, from your parents to your mentors, that have influenced you in this. **But if you could talk to a young chemist today, what would you tell them in terms of how to approach others? What is the key to being successful, and to drag your chemistry into different directions?**

**Kevan:** There's an infinite number of things you could do with your time and science. And I find the best way to collaborate with people is when there's an ease of interaction, because when we get deep into the science, as you and I did, there are always times when the data don’t line up. You also want to be very curious about the biological question. I could have a lot of things seem right in a collaboration, but if the biological question is too far away from the chemistry, I just won't have the same motivation.

**Ross: Have you actually declined or walked away from a collaboration?**

**Kevan:** We've had projects in the lab where a student really wanted to pursue it, and I have agreed as it sounded like it was going to be close to what we do. If the student is great, they take it on, but if the project gets layer upon layer away from the chemistry, it gets harder. Then, because it's a student in my lab, I try to bring in another collaborator that I know has the right expertise. That part is fun, but I'd rather know at the beginning roughly the parameters of the project.

**Ross:** I know that you're expanding into other arenas; PINK-1 with Parkinson's and even COVID. **In your lab, do you decide to work on any interesting questions or do you try to rein in your lab?**

**Kevan:** I try to just work on interesting questions. I don't try to say what we will or won't do. Working on COVID was amazing, but I think when you come from chemistry, there are certain holes in your knowledge. I think the most important thing is to always ask the question that's on your mind because nobody knows everything, and this helps you to make sure you don't have gaps.

**Ross:** You have a willingness to plunge in and take on challenging problems, which I know people, such as Frank McCormick, have pushed you towards.

Your specialty, at least initially, was kinase inhibitors. During that time, the ‘bump-and-hole’ method for identifying targets was really powerful, and very clever. But in what may turn out to be one of your most-impactful identifications, you developed the first covalent inhibitor of KRAS [the most commonly mutated oncogene, associated with poor responses to standard cancer therapies], specifically with the G12C mutation. KRAS was known for so long as being one of the ‘undruggables’ and was called the Death Star of cancer targets. Excitingly, sotorasib [the compound inspired by your breakthrough] has recently been approved [for adult non-small cell lung cancer patients with KRAS^G12C^]. So, a broad congratulation is in order but, I think, what is equally amazing is how fast everything happened. You published your original paper in 2013; eight years later, add COVID, and now you have an approved therapy. **Were you surprised with how quickly things moved? And why do you think things moved so quickly?**

**Kevan:** There were a lot of pieces that contributed to it happening quickly. When we published our paper in 2013, people were already coming up with molecules that could bind to RAS. In 2012, there was a paper from Genentech (Maurer et al., 2012) and another from Stephen Fesik at Vanderbilt University (Sun et al., 2012), where they had done a screen using nuclear magnetic resonance spectroscopy and found a little pocket [in the RAS 3D structure] and a small molecule that would bind, but it was weak with low affinity. Then Nathanael Gray's lab at Harvard described a compound that could inhibit RAS^G12C^ (Lim et al., 2014), which was a covalent molecule based on the structure of GTP. At this point, we were getting ready to publish our approach – this other pocket we had identified in RAS and called the switch-II pocket, which was distinct from the others (Ostrem et al., 2013). Our molecule that bound in this pocket also pretty weak, although we saw the beginning of cellular activity. I think what really saved it and made it progress very fast was that I started working with Yi Liu, Pingda Ren and Troy Wilson, and we started a company called Araxes. They just launched into optimization and published a paper in 2016 that showed the first really good molecule with potent cellular activity (Patricelli et al., 2016). Then a couple of years later they published a beautiful active molecule that worked *in vivo* (Janes et al., 2018). If we had just published the 2013 switch-II paper and had not started a company, I think pharma would have looked at the paper, played around with the compound, and not taken it further. That was the period when people at companies thought there was a reproducibility crisis in academia. I've heard from pharma companies that they took our paper, synthesized every single key molecule and reproduced our activity – but didn't see developable activity. However, because I had a great relationship with Yi and Pingda, they believed in this molecule and optimized it. Now, the scaffold they discovered is in the core of every other G12C inhibitor. So, to me, the fast part was really credited to them. Also, RAS-mutant tumours are very ‘RAS addicted’ and once there was a chemical that you could target it with, the drug progressed very fast, which was also the case for inhibitors of viral Src and BCR-ABL1.

**Ross:** That's really interesting because your job is to solve the RAS problem and, in part, you did that by establishing or encouraging a company to help drive it forward.

The molecule shows efficacy for months but not years, which is fairly typical for precision medicine molecules. My lab focuses on this as well, so I'm obviously a believer – but it's a little frustrating that the patient doesn't get a cure. **What do you think is the ceiling for drugs, in general? And how are we going to break through that initial ceiling?**

**Kevan:** I think this is, right now, the key question in oncology. If we just keep making drugs that give us six months extra [survival], that isn't the real breakthrough. To me, a cure would be a patient has cancer, gets treatment and, when they pass away, it's not because of their cancer. For example, my colleague Neil Shaw told me that ∼30% of [chronic myeloid leukaemia] patients treated with BCR-ABL1 inhibitors can be cytologically converted to be tumour free. Even when there are patients that can be cured, we still don't quite understand why 70% of patients aren't. I think we need better molecules, like inhibitors that drive the signalling down to ∼2% of the existing [level]. So that, to me, is one answer.

The other answer is molecules that are more specific for the mutant and don't inhibit the wild type. As we know, that's a challenge. The G12C drug is great because it's only inhibiting the mutant, which is only present in the tumour. This inhibitor is now a backbone for many [drug] combinations that, I think, will lead to some cures; because when we get the right combination, there should be a profound response.

“We've got a lot of six-month treatments, which is great, but now let's go for a functional cure.”

And then, of course, starting treatment earlier. We've got to start it before [the cancer cell accumulates too many additional mutations], to target the truncal mutants. I think [this ‘cure’] is going to come in the next five years, certainly in ten years – but it's going to require chemistry, a drug combination and early detection. Early detection seems like a panacea, but I don't want us to put all our eggs in that basket. I think we should be focusing on getting earlier therapy and the right molecules. We've got a lot of six-month treatments, which is great, but now let's go for a functional cure.

**Ross: Do you think that your research is in the right chemical space and you just need to mature it? Or do you think there's going to be a need for new scaffolds? Also, while we're talking about this, I'll throw in polypharmacology [when a drug has multiple targets or disease pathways], which itself is somehow controversial. So maybe talk a little bit about all of these.**

**Kevan:** I think when you get a target, you can swap in and out scaffolds. The question is, can you get durable and potent inhibition, which can come from a brand-new screen. That's how Plexxikon and Genentech came up with vemurafenib, the breakthrough RAF inhibitor. They started with sorafenib but took a step back and said “Let's start with tiny scaffolds at Plexxikon, find a starting point and then grow it out”, which is the basis of fragment-based drug discovery and probably the biggest breakthrough in drug discovery in the last 20 years. It's a very powerful way to get the most drug-like, low-molecular-weight and highly ligand-efficient molecule, all of which are properties of very good drugs. Now there's also the covalent approach that helps achieve more-potent and full inhibition, and there's the Proteolysis Targeting Chimeras (PROTACs) approach, which degrades proteins. What is awesome is that, every way you perturb a biological system, there's a difference between getting rid of the protein, and sticking a drug in its active site and leaving the scaffold there. I think the molecular glues have opened people's eyes to bringing [two proteins] together, which even without their degradation can create a signal to the cell. And that, I think, is going to be a very big, continuing and emerging area of research.

“[…] when there's something special about a molecule, it's probably a polypharmacological property, and finding the special molecules is what drug discovery is all about.”

And when you say polypharmacology, I really think that's how drugs are special – not only because we worked together on this so much and believe that it has such a promise. I think it's also worth keeping in mind that, when there's something special about a molecule, it's probably a polypharmacological property, and finding the special molecules is what drug discovery is all about. It's great to see more biological thinking around a special molecule and people are digging to find out why it is special. I've heard some talks recently about old kinase inhibitors; and now that we have many of them, you can see which one is special and figure out why it's special. I think there's going to be another surge of polypharmacology coming, which should be really interesting.

**Ross: So, if we were to have this conversation again in 10 years, where do you hope RAS research will be?**

**Kevan:** The immediate answer is molecules that will inhibit each of the alleles. I think these already exist but are hidden in companies. The cysteine (G12C) has already been approved, but the aspartate and the valine variants would be next. Then even the esoteric, the rarer ones, we've already got. Once we get all that chemical matter, I think there will be another group of molecules that will emerge; maybe an ERK inhibitor, a MEK inhibitor, a RAF inhibitor or a SHP2 inhibitor. I feel like that's the second wave of small-molecule research.

Then I think the immune–oncology connection will become important because RAS is such a good suppressor of the immune system. Now that we've got agents to eliminate that suppression, the checkpoint inhibitor therapies will probably work. G12C lung tumours are typically caused by smoking, meaning they've got a high mutation burden and are immunogenic. But G12C pancreatic cancer does not have a particularly high mutational burden, so the checkpoint inhibitors may not work [as efficiently]. There's just beautiful work from Bert Vogelstein, Drew Pardoll and Ken Kinzler who've made antibodies against the KRAS-mutant peptide on MHC, they call them MANAbodies. I don't know right now what the next ten years are going to show, but I hope this progress comes in just five years.

**Ross:** We can both agree that it's likely we don't know about it yet. So, beyond RAS and beyond kinase inhibitors, **what's on the horizon for the Shokat lab?**

**Kevan:** We made a molecule a few years ago to bypass mTOR inhibitor-resistance mutants, which has a bivalent nature. We call it a bitopic inhibitor and it can bind an allosteric and an orthosteric site. Revolution Medicines optimized it and it is now being tested in the clinic. But this compound's high molecular weight [complicates its entry] into cells. So now we're working on better understanding of how molecules actually get into cells. Is it an uptake receptor? Is it passive permeability? Is it avoiding efflux? We know a lot of those things but, actually, there are still a lot of mysteries. With my UCSF colleague Keith Yamamoto, we always come back to the question of how glucocorticoids get across the cell? If we could get a better understanding of how bigger molecules get across, I think that opens up new targets.

And then, the other thing I want to think more of is a brand-new mode of recognition, which is not recognising the three-dimensional shape of proteins but recognising their primary sequence. We've got siRNA, we've got CRISPR, we've got base editors, so we can deal with linear sequences in the nucleotides. A good friend, Aseem Ansari, has beautiful programmable polyamides from Peter Dervan's lab, which recognise the minor groove in a sequence-specific way. People have worked on sequence-specific linear recognition in DNA and RNA for many years but, in proteins, we've always used three-dimensional lock and key recognition. There was a molecule that was discovered at Pfizer in a cell-based screen for blocking a certain cardiovascular target called PCSK9. Out of 2 million molecules, they found one that blocked its production but they couldn't figure out what it was doing. They found out that it bound and blocked 18 sequences out of 2000 in the exit tunnel of the ribosome, while the PCSK9 nascent chain was going through. It's a pretty stunning thing. We know from the antibiotic world that there are natural products that do that, but this was the first synthetic example of that. So, I would like to explore that more, because anything that gets you a completely new target recognition, really opens a lot of doors.

**Ross:** One of the things I love about chemistry as a chemistry-adjacent person is that, much like genetics and genetic screens, there's a bottom line and a hypothesis-generating aspect to what you're describing.

So, to shift towards advice for younger scientists, **how is the chemistry field different today and what are the characteristics of a successful 2021 chemist?**

**Kevan:** That's a great question to think about. In the context of chemical biologists, many of the same challenges we had still apply. You either need to come up with a molecule that will do ‘X’ or you need to be comfortable making hundreds of molecules really fast if you come from synthetic expertise; or, if you're more from the biological side, you'll make combinations to gain a new function. But now, there's a growing appreciation of what to work on that is relevant to both drug discovery and biological impact. The sophistication has gone up so much.

**Ross:** I know this is true in my world, which is that we had to physically make a lot of stuff when we were at the bench back in the day – but now you can just order it. There are so many kits that you can order, just like in molecular biology. So, **I wonder if there's more of an emphasis now on the big picture because you can focus more on the question.**

**Kevan:** Now, there should be a lot more time spent with new students on strategy. Because, you're right, you don't need to spend all that time expressing and purifying Taq polymerase like we used to do. The lab would just buy so many reagents. You could think of an experiment, go on the computer, order it and, two days later it would come. But with the COVID-19 pandemic, we didn't have much time in the lab because there was a restriction on density. I felt like everything got drawn out. So, I thought, what if we just flip it around and everybody in the lab comes up with an experiment that they can do with reagents that are already in our lab. That really prompted a lot of creativity. Something, from two months ago, that was parked could be combined with something else – and it was a really good thing for us to try.

Also, there's now much more availability of supplementary information buried in tables and papers, for example, from CRISPR and RNAseq experiments. It feels like there's a wealth of information, but it's how you connect it and find the shortest path to an interesting answer.

**Ross:** You know, artists often say that they get most creative when you apply constraints to them. **Did it work? Did you come up with some cool stuff?**

**Kevan:** There were ideas that were just sitting there, and it gave us something that wasn't the original goal but was a step forward that we didn't have before. That constraint thing dawned on me 10 years ago, with RAS as a perfect example. Whenever I would talk with Frank [McCormick] about RAS, he'd say “It's an enzyme that's broken and stuck, loaded with GTP”. There were always things that he, Fred Wittinghofer, Roger Goody and others had uncovered, that just seemed like walls that you couldn't get over. And it dawned on me that this constraint was actually a great way to focus. I think in chemical biology, you can always do something else, but in biology and disease there's only one thin lane, and you have to stay in that. That constraint is what I'm really after now, and making sure that you maximise the creativity within the constraints. We know that from cancer because, in the case of RAS, it's not going to go away until we find a drug for it.

**Ross:** Incidentally, I know Frank, he's a good guy and can be very persuasive. **How did he persuade you to work on RAS?**

**Kevan:** I listened to him twice. When I first came to UCSF, he said “Kevan, PI3K is highly mutated and it's an important oncogene”. So, we started working on it and a student, Zack Knight, had a beautiful set of papers uncovering new molecules, with some either being tested (sapanisertib) or approved (duvelisib) for use in the clinic. And I thought that Frank really knows his targets because, before we started on it, PI3K was not that apparent. But all those years working on it, from 2002 to 2010, Frank kept saying “Remember RAS, that's the one”. And it worked in the end because he kept inviting people like Fred Wittinghofer and Roger Goody, who were both at the Max Planck Institute in Dortmund (Germany) and had done incredible experiments to investigate the structure, biochemistry and cell biology of RAS. My colleague Jack Taunton was working on irreversible inhibitors, so cysteines were always around, and Jim Wells had a tethering library. So, I felt it was a lucky chance to put all these pieces together at the right time.

**Ross: So, Frank created a virtual community?**

**Kevan:** Yeah, and now he's doing it at the National Cancer Institute.

**Ross:** Your family's been very important to you. I know your parents, for example, were very politically active, and I know you and [your wife] Deborah have also been pretty active over the years. **So, where do you see the role of scientists in today's public discourse?**

**Kevan:** With the pandemic, it's so frustrating to have this unbelievable vaccine technology emerge so quickly – although, we all know it took a long lead time – but then to have it sitting there and have it become political football. I'm struggling with how this mountain of discoveries and these vaccines have been developed but then have some people look at it and say “There's no mountain there, don't use that.”

“I love telling people about discoveries and how they're made, and I think sometimes we can get through to people because it's not so foreign if you break it down in an interesting way.”

My daughter Leila just graduated from Reed [College] and she's going to do a master's, hopefully, in Edinburgh, Scotland, within science and communications. She did a great thesis on the ethnography of the COVID-19 research group based here at UCSF, called QBI Coronavirus Research Group (QCRG), and how people communicate confusing results. So, I feel like the more we can communicate how science is done, we can help the public understand. I love telling people about discoveries and how they're made, and I think sometimes we can get through to people because it's not so foreign if you break it down in an interesting way.

**Ross:** I wonder if this would go towards your advice to young scientists. **I wonder if, as we train scientists, we could fill in the gap between our generation of scientists and your daughter's, in terms of science communication?**

**Kevan:** The one thing I try to do is get the students and postdocs in the lab to give talks where they don't dive deeply into the tiny parts of their project but give people a better perspective. And I feel like, if we do that amongst ourselves, then we're going to have the ability to explain it to the public. Deborah [who is a physician] and I did a thing with our families a few months ago about COVID and the vaccine. We presented a set of slides on Zoom, and it was great. The fantastic questions people, who don't do science, come up with never cease to amaze me – when you explain the science to them in normal language. When one of my students graduated, his parents came to listen to his talk, and then we came upstairs to the lab for the party. And I showed them the crystal structure of a protein from his project with three dimensional glasses. And I will never forget, Chris's mother looked at it and she said “Well, that looks like a pocket over there, why doesn't he make a molecule that fits there.”; and I said “I told him to do that.”

**Ross:** That's pretty amazing. **So, if not science, what would you do?** I know you were into sports when you were younger.

**Kevan:** I really don't know. I wanted to go to medical school when I was an undergrad, but I didn't get admitted. But sometimes not getting admitted is the best gift because there are so many other things to do. So, I actually don't know where I would go if you took science away. I definitely love sports, but I don't know if I could be a professional cyclist because I like to eat, so that wouldn't work.

**Ross: So, is there any question that you think I should have asked that you would like to answer or anything you'd like to add?**

**Kevan:** The first question you asked had many parts, and one part I didn't answer was how genetics and pharma interact. This gets to the core of why pharma companies work on what they do, and how biological insights come together there. It really reminds me that there's often a disconnect between biology and drugs. And there doesn't always have to be. In treating your fly models with the drugs that we've worked on together – they often really line up. But there's always the chance for a disconnect between genetic and pharmacological targeting. In my career, starting back at Princeton, that was such a profound thing because we expect things to line up; and when they don't, we think one of them is a useless piece of information. It's really the flexibility of being able to look at a biological experiment and a pharmacological experiment, hold the two results in your head and not be uncomfortable when they don't just beautifully line up but, rather, look into the discrepancy and then evolve that. That's what Pharma very rarely can do, although they're beginning to see the importance of this approach. But I just love when that dawns on people because that's when they're really understanding things.

**Ross:** I can certainly amplify that. Sometimes with a biological problem, I don't understand why the chemical rules would win over the biology.

**Kevan:** There's a lot of good discoveries to be made in those areas.

## References

[DMM049468C1] Janes, M. R., Zhang, J., Li, L.-S., Hansen, R., Peters, U., Guo, X., Chen, Y., Babbar, A., Firdaus, S. J., Darjania, L. et al. (2018). Targeting KRAS mutant cancers with a covalent G12C-specific inhibitor. *Cell* 172, 578-589.e17. 10.1016/j.cell.2018.01.006 29373830

[DMM049468C2] Lim, S. M., Westover, K. D., Ficarro, S. B., Harrison, R. A., Choi, H. G., Pacold, M. E., Carrasco, M., Hunter, J., Kim, N. D., Xie, T. et al. (2014). Therapeutic targeting of oncogenic K-Ras by a covalent catalytic site inhibitor. *Angew. Chem. Int. Ed Engl.* 53, 199-204. 10.1002/anie.201307387 24259466 PMC3914205

[DMM049468C3] Maurer, T., Garrenton, L. S., Oh, A., Pitts, K., Anderson, D. J., Skelton, N. J., Fauber, B. P., Pan, B., Malek, S., Stokoe, D. et al. (2012). Small-molecule ligands bind to a distinct pocket in Ras and inhibit SOS-mediated nucleotide exchange activity. *Proc. Natl. Acad. Sci. USA* 109, 5299-5304. 10.1073/pnas.1116510109 22431598 PMC3325706

[DMM049468C4] Ostrem, J. M., Peters, U., Sos, M. L., Wells, J. A. and Shokat, K. M. (2013). K-Ras(G12C) inhibitors allosterically control GTP affinity and effector interactions. *Nature* 503, 548-551. 10.1038/nature12796 24256730 PMC4274051

[DMM049468C5] Patricelli, M. P., Janes, M. R., Li, L.-S., Hansen, R., Peters, U., Kessler, L. V., Chen, Y., Kucharski, J. M., Feng, J., Ely, T. et al. (2016). Selective inhibition of oncogenic KRAS output with small molecules targeting the inactive state. *Cancer Discov.* 6, 316-329. 10.1158/2159-8290.CD-15-1105 26739882

[DMM049468C6] Sun, Q., Burke, J. P., Phan, J., Burns, M. C., Olejniczak, E. T., Waterson, A. G., Lee, T., Rossanese, O. W. and Fesik, S. W. (2012). Discovery of small molecules that bind to K-Ras and inhibit Sos-mediated activation. *Angew. Chem. Int. Ed Engl.* 51, 6140-6143. 10.1002/anie.201201358 22566140 PMC3620661

